# An accessory muscle belly or an accessory muscle head? An unusual arrangement of muscles in the anterior compartment of the forearm

**DOI:** 10.1007/s00276-023-03084-0

**Published:** 2023-01-25

**Authors:** Klára Gabríková, David Kachlík, Miroslav Belbl, Vojtěch Kunc

**Affiliations:** 1 Department of Anatomy, Second Faculty of Medicine, Charles University, Plzenska 130/221, 150 06 Prague 5, Czech Republic; 2 Department of Health Care Studies, College of Polytechnics, Tolsteho 16, 586 01 Jihlava, Czech Republic; 3 Clinic of Trauma Surgery, Masaryk Hospital, Socialni Pece 3316/12A, 400 11 Usti nad Labem, Czech Republic

**Keywords:** Flexor pollicis longus muscle, First lumbrical muscle, Accessory muscle head, Musculotendinous interconnection

## Abstract

**Purpose:**

Knowledge of the unusual arrangement of the flexor pollicis longus (FPL) muscle is important as the variable tendon may be a rare cause of carpal tunnel syndrome.

**Methods:**

During a routine dissection at the Department of Anatomy, an unusual formation of the FPL muscle was observed in a formalin embalmed Central European cadaver.

**Results:**

This report presents a variation of the FPL muscle, where the muscle split and formed a separate accessory head inserting into the first lumbrical muscle. Moreover, a tendinous interconnection was present between the FPL muscle tendon and the tendon of the aberrant muscle head.

**Conclusion:**

The cases described by previous literature, concerning the Linburg–Comstock variation or the accessory head of the first lumbrical muscle originating from the FPL muscle, are closest to the present case. Such variation has a clinical significance ranging from the functional limitation of the thumb and index finger movement to the potential median nerve compression.

## Introduction

Lumbrical muscles (lumbricals; *musculi lumbricales*) belong to the intrinsic musculature of the hand. Usually, the first and second lumbricals are unipennate and arise from the lateral (radial) sides of the flexor digitorum profundus muscle (FDP) tendons for the index and the middle fingers. The fourth and fifth are usually bipennate and arise from adjacent aspects of the FDP muscle tendons for the middle and ring fingers, and ring and little fingers, respectively. Narrow tendons of the lumbrical muscles get around the lateral side of the metacarpophalangeal (MCP) joints and insert into the dorsal digital expansion of the dorsal aponeurosis through the lateral bands of the respective finger. The role of the lumbrical muscles is to flex the MCP joints and to extend both proximal (PIP) and distal (DIP) interphalangeal joints of the fingers. Their function is essential for precise movement of the fingers.

Flexor pollicis longus (FPL) muscle belongs to the deep musculature of the anterior compartment of the forearm. The FPL muscle originates from the anterior surface of the radius, between the insertion of the pronator teres muscle and the insertion of the pronator quadratus muscle and abounding interosseous membrane of the forearm. It courses distally through the carpal canal, laterally to the tendons of the FDP muscle, and inserts onto the base of the distal phalanx of the thumb. The function of the FPL muscle is to flex the MCP and IP joints of the thumb.

We report a case where the FPL muscle split and formed an extraordinary portion that represented the accessory muscle head of the first lumbrical muscle (L1). There have been described several similar cases reporting the additional (accessory) origin of the L1 [1, 12, 16]. However, any of these situations do not accurately match the presented case. The greatest similarity can be seen among the cases, describing the Linburg–Comstock variation (LCV) composed of the musculotendinous interconnection (a muscle portion arising from the FPL muscle and forming a separate tendon that joints the FDP muscle tendon [7, 10, 11, 19], or that describes the accessory head of the L1 from the medial aspect of the FPL muscle [11, 17–19]. Clinical relevance comprises the potential impact on the functional limitation of the thumb and index finger movement. Furthermore, people with an aberrant tendon within the carpal canal (CC) are more likely to develop the carpal tunnel syndrome [1, 7, 9–11, 17, 19].

## Case report

A variable muscle head of the first lumbrical muscle was observed unilaterally during the routine dissection of the left forearm of an 82-year-old female, formalin embalmed, Central European cadaver.

**Fig. 1:**
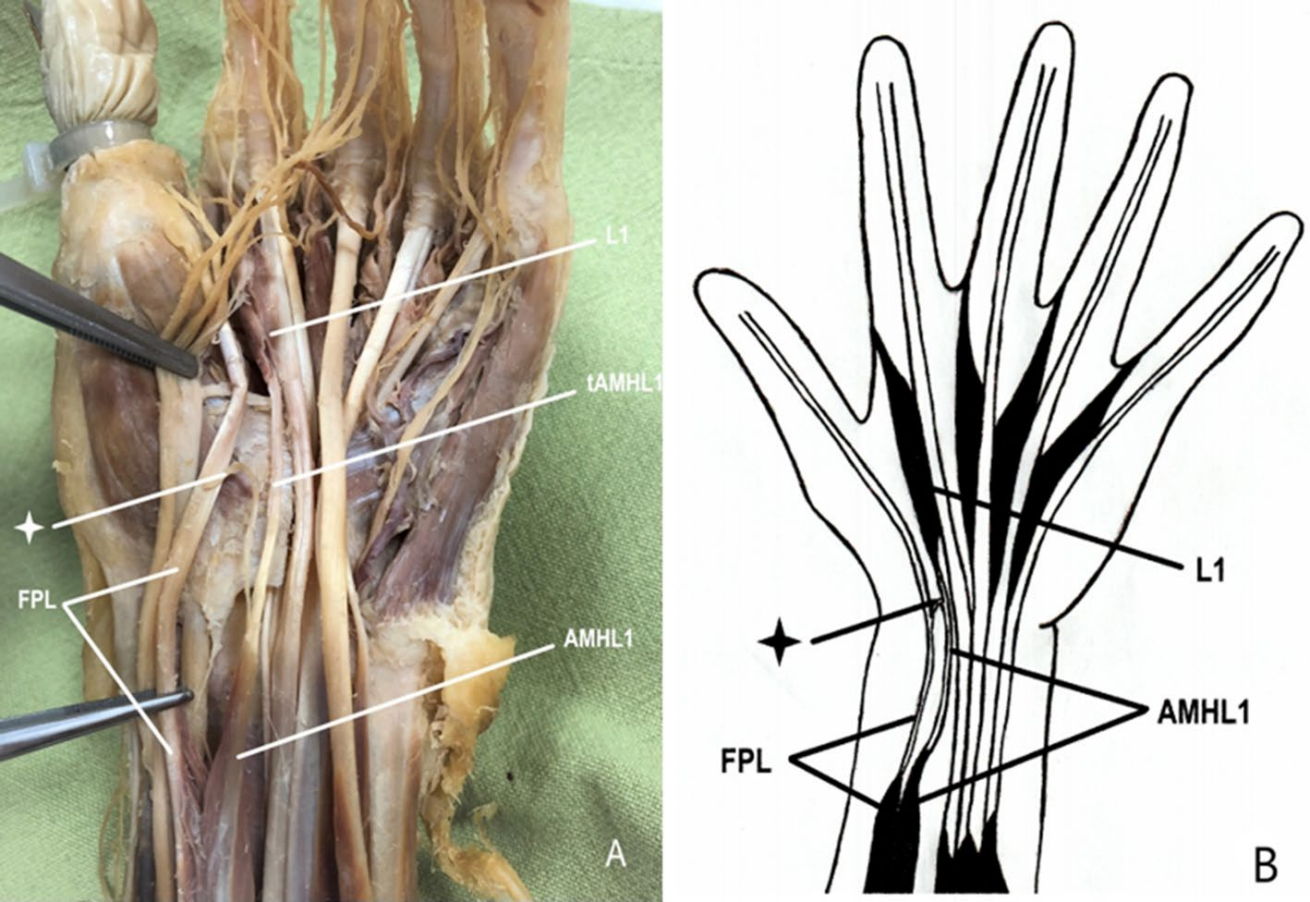
**A** The accessory muscle head derived from the FPL muscle joining the regular muscle belly of the L1 muscle. **B** Scheme of the anomalous muscle head of the L1 muscle. *L1* first lumbrical muscle, *tAMHL1* tendon of the accessory muscle head of the first lumbrical muscle, *AMHL1* accessory muscle head of the first lumbrical muscle, *FPL* flexor pollicis longus muscle, *Asterisk* tendinous interconnection between the FPL muscle tendon and the aberrant tendon

The accessory muscle head derived from the FPL muscle and joined to the regular muscle belly of the L1. The FPL muscle originated from the anterior surface of the radius and abounding interosseous membrane of the forearm in the standard fashion. Its tendon passed via the carpal canal, lateral to the FDP tendons, and inserted onto the base of the distal phalanx of the thumb. Proximally to the carpal canal (80 mm proximal to the proximal margin of the flexor retinaculum), the accessory head separated from the regular FPL muscle and gave rise to a separate aberrant tendon (39 mm long), which descended towards the carpal canal beneath the median nerve, medially to the FPL tendon and laterally to the FDP muscle tendon for the second finger. Then, it reached the L1 muscle 15 mm distal to the carpal canal. The accessory muscle head received its nerve supply from the motor branch of the anterior interosseous nerve. (Fig. 1).

Besides this anomalous origin, the L1 muscle arose according to a norm from the lateral aspect of the first FDP muscle tendon and inserted into the dorsal digital expansion of the dorsal aponeurosis of the index finger. Nerve and blood supply were provided by a branch of the median nerve and the superficial palmar arch, respectively.

The proportions of the accessory muscle head and its tendon (inserting into the L1) were comparable with those of the regular FPL muscle belly. The length and width of the accessory muscle head were 167 mm and 8 mm, respectively. The width of its tendon was 2 mm, those of the regular FPL muscle belly were 160 mm, and 7 mm, the width of its tendon was 3 mm.

Moreover, a tendinous interconnection was present between the FPL muscle tendon and its aberrant tendon. The position of the interconnection was within the carpal canal beneath the median nerve. Its length was 120 mm and width 1 mm.

No other anatomical variants were observed in the forearms or hands of the cadaver.

## Discussion

The lumbrical muscles are a very unsteady component of the intrinsic musculature of the hand, comprising a wide range of diversity concerning both the origins and the insertions. The multifariousness of these muscles begins from the unipennate or bipennate nature. The first and second lumbrical muscles may occur bipennate, whereas the third and fourth may vice versa be unipennate [12]. The absence [12, 18] of some of the lumbrical muscles has been described as well as the presence of an accessory [8, 12] or proximal [1] origin from the tendons of the adjacent muscles (FDP, FPL, flexor digitorum superficialis (FDS)) either from the forearm or hand. Also, muscle hypertrophy associated with genetic predispositions [1] or acquired conditions [1] has been reported. Among the most common variations of the insertion belong a misplaced or split insertion and insertion on the proximal phalanx. Insertion of the lumbricalis muscle is called “misplaced” if it inserts on the medial side of the corresponding finger and “split” if it inserts on both the lateral and medial sides of the corresponding fingers [12].

Innervation of the lumbrical muscles may also differ from the usual pattern. Concerning the L1, there were described two cases of the L1 innervated by both the median and the ulnar nerve by Hur and another case reported by Mehta and Gardner [4, 12].

Nevertheless, deviations of the L1 muscle are the rarest [12], compared to the variations of the other lumbricals. According to Mehta and Gardner, the variations concerning the L1 muscle occur in 17.3%, whereas those of the L2 muscle have been encountered in 32% (commonly in its accessory origin or bipennate formation); the L3 muscle has been variable in 54.7% (variations are usually involved in its insertion as a “split insertion”), and the L4 muscle in 40% (as the most frequently absent muscle compared to all others) [12]. The most prevalent variations of the L1 muscle comprise the accessory origins within the hand, usually from the FDS tendon for the index finger or from the FPL tendon. However, the accessory muscle head from the forearm is less frequent [12] and its origin may vary according to different studies. Wood described the accessory muscle heads arising either from the middle third of the anterior surface of the radius or from the accessory tendon dividing from the FPL muscle and joining the FDP muscle for the index finger [18]. Kopuz et al. observed its origin from the lateral part of the interosseous membrane of the forearm and the anterior surface of the radius [8]. Afroze recorded four cases of an accessory muscle head from the distal belly of the FDS muscle [1] and Sawant described the origin from the lateral side of the FDP tendon for the index finger [16]. Even the separate portion originating from the dorsal aspect of the FDS muscle was observed as an aberrant muscle head [8]. Any anatomical variation of these muscles can have a crucial impact on hand mobility as it can interrupt, limit, or modify the full range of proper muscle function [13].

The most prevalent variations of the FPL muscle include the accessory muscle head that joins the FPL muscle or the tendinous interconnections with the neighboring muscles. The most frequently described variations are the accessory head of the FPL muscle (one of the variants of Gantzer’s muscle) [2, 5, 6, 15] and the Linburg–Comstock variation [7, 10, 11, 19]. According to Asgharet al., the prevalence of the Gantzer’s muscle concerning only the accessory head of FPL is 48% out of the total 65%. [2]. The prevalence of the Linburg–Comstock variation is moving around 21% as reported by Yammine [19]. However, the accessory muscle belly arising from the FPL is less common [17, 21].

The Gantzer’s muscle (GM) is described as an accessory head of the FPL muscle, or less prevalent of the FDP muscle, occurring rather bilaterally than unilaterally. It arises from the medial epicondyle of the humerus, the coronoid process of the ulna, or both (either alone or via fibers from the FDS muscle), or the FDS muscle. Nevertheless, the origin of the muscle fascia is somewhat unique. Its tendon joins with the FPL muscle tendon (most often within the proximal third of the forearm) or with one of the FDP muscle tendons (usually at the wrist level), or more rarely as a bifid tendon with both the FDP and the FPL muscle tendons. Its pattern and prevalence are very heterogeneous in conformity with many papers [2, 3, 5, 15]. The highest rate is among the American population; however, among Europeans, its prevalence decreases. The clinical importance is the potential risk to cause compression neuropathy of the anterior antebrachial interosseous nerve, known as Kiloh-Nevin syndrome, or even of the median nerve trunk, as the muscle usually lies among these two [5, 15].

Linburg–Comstock variation (LCV) is a frequent congenital variant tendon slip stretching between the FPL and the FDP muscles, occurring rather unilaterally than bilaterally. The tendinous, or less prevalent musculotendinous or fibrous interconnection unites the FPL muscle tendon with one of the FDP muscle tendons (most often the first tendon (for the index finger), or less commonly other three (for the middle, ring, or little finger)). The cases describing the tendinous split or even musculotendinous portion from the FPL muscle to the L1 muscle are much more unique. A wide range of its pattern exists in the location as well as the proportions. The importance of such variation may be seen in the impact on the separate precise thumb and fingers movement [10, 11, 20]. The persons with the LCV are unable to flex the MCP joint of the thumb without synergic flexion of the distal IP joint of the index finger (or/with the distal IP joints of the other involved fingers). This condition may stand as a limiting factor for some professions, such as musicians dependent on precise hand movement [7, 19]. Clinical significance may be observed in the higher tendency to develop tendosynovitis by the presence of the variant tendon slip. The treatment in this case is a surgical intervention for the removal of the interconnection [7, 10, 11, 19].

In comparison with the two above-mentioned variations, the accessory muscle belly of the FPL muscle is much more unusual. In one case report by Swamy Shantakumar et al., the muscle portion arising from the FPL muscle formed three aberrant tendons, two merging with the deep surface of the flexor retinaculum, the third fusing with the FDS muscle tendon. In this case, the accessory origin of the bipennate L1 muscle arose from this third aberrant tendon [17]. Another case reported by Zielinska et al. described six accessory heads arising from the FPL muscle and adjacent interosseous membrane of the forearm. All of them merged and fused with the FPL tendon [20]. The incidence of the bifid FPL muscle linking with the FDP muscle tendon is a bit more frequent; however, it has to be viewed as one type of the LCV [11, 15].

If we consider an *accessory muscle head* to be a muscle portion connecting the corresponding muscle and originating from non-muscular structures of the forearm (if we do not count with the rare origin of the GM from the FDS muscle), and an *accessory muscle belly* to be a muscle portion arising from the corresponding muscle and joining the tendon of another muscle, our case describes either an accessory muscle belly of the FPL muscle with the unusual insertion to the L1 muscle or an accessory muscle head for the L1 muscle with an unusual origin from the FPL muscle. The most comparable situations to ours can be seen in some LCV cases describing the musculotendinous interconnection deriving from the FPL muscle and reaching the L1 muscle instead of fusing with the FDP muscle tendon [7, 10, 11, 19, 20]. Mehta and Gardner reported an almost identical condition with an accessory origin of the L1 muscle from the medial border of the FPL muscle [12]. Another similar situation may be found in the papers by Wood, where the accessory head originating in the middle of the anterior surface of the radius reached the L1 muscle without any pure connection with the FDP muscle tendon [18].

The tendinous interconnection between the aberrant tendon and the FPL muscle tendon has probably little clinical significance. As there is no connection with the FDP, the traditional LCV test may be negative. A similar situation may occur even when the connection with the FDP is presented. Prasatkaew et al. describe a case of reversed LCV, where the LCV test comes out fault negative. However, it is possible to test the presence of the interconnection by switching the role of the fingers (actively flexing the distal interphalangeal joint of the index and observing the flexion of the thumb) [14]. In our case, it is not possible to test the presence of the interconnection and the reason is probably a common muscle base.

Such an unusual arrangement can be an outcome of inadequate embryological development. During the early stage of growth, the flexor mass divides into superficial and deep layers. The deep layer gives rise to the flexor carpi ulnaris muscle and then divides to form more superficially located FDS muscle and beneath it lying FDP muscle, from which the FPL muscle splits off [3]. However, the process of separation does not always occur properly. Due to these circumstances, variant muscle bellies, heads or interconnections can appear among flexor muscles [5, 15, 17]. Incomplete cleavage of the flexor mass during embryological development is, in all probability, the cause of the presence of accessory tendinous interconnections linking the FPL muscle tendon and the aberrant tendon, presented in our case, as well as the accessory L1 muscle head.

An accessory tendon, present within the carpal canal, has a significant clinical relevance as it increases the canal’s contents. Consequently, it may be associated with compression neuropathy of the median nerve. The surgeons should be aware of such unusual arrangements, while operating in this area during the surgical intervention to treat carpal tunnel syndrome [7, 10, 11, 17, 19].

## Data Availability

All further data are available upon request to the corresponding author.
